# Exploring the mediating role of psychological resilience between social support and anxiety in nurses: a cross-sectional study in Chengdu, Sichuan, China

**DOI:** 10.3389/fpubh.2026.1765061

**Published:** 2026-02-23

**Authors:** Junyi Hou, Mingzhu Song, Hui Li, Xinlin Miao, Xia Wang, Yesha Liu, Jianfeng Sun, Chan Huang

**Affiliations:** 1 School of Preclinical Medicine & School of Nursing, Chengdu University, Chengdu, China; 2 School of Acupuncture and Moxibustion, Affiliated Hospital of Chengdu University of TCM, Chengdu, China; 3 Hospital of Chengdu University of Traditional Chinese Medicine (TCM Hospital of Sichuan Province), Chengdu, China

**Keywords:** nurses, anxiety, mental health, psychological resilience, social support

## Abstract

**Background and aims:**

Nurse anxiety is a significant public health issue that impacts individual well-being, healthcare system stability, service quality, and patient safety. This study aimed to assess anxiety levels among Chinese nurses and to investigate the association between social support and anxiety, specifically examining the potential mediating role of psychological resilience. The findings are intended to enhance the understanding of mental health mechanisms and inform targeted interventions for nurses.

**Methods:**

A cross-sectional online survey was conducted from February to April 2025 with 1,133 nursing staff in Sichuan Province, China, using stratified and snowball sampling. Participants completed the Self-Rating Anxiety Scale (SAS), Social Support Rating Scale (SSRS), and Connor-Davidson Resilience Scale (CD-RISC). Data were analyzed using *t*-tests, ANOVA, Bayesian Estimates, Pearson correlation, and bootstrap method. The hypothesized mediation model was tested using structural equation modeling (SEM) with latent variables.

**Results:**

A total of 35.7% of nurses reported experiencing anxiety. Both social support (including its sub-dimensions) and psychological resilience (including its sub-dimensions of self-efficacy, hope, and optimism) showed significant negative correlations with anxiety. Mediation analysis confirmed that psychological resilience partially mediated the relationship between social support and anxiety, accounting for 21% of the total effect. The pathway mediated by psychological resilience was strongest for subjective support, with an indirect effect constituting 41% of its total impact. In SEM with latent variables, the mediating effect of psychological resilience on the relationship between social support and anxiety remains statistically significant after separating and controlling for measurement errors.

**Conclusion:**

The research points to the interaction between social environment (support) and individual characteristics (resilience) as a key determinant of nurses’ emotional regulation. This implies that strategies to mitigate anxiety should prioritize cultivating a supportive climate and promoting the functional use of social resources, which in turn bolsters psychological resilience and overall mental health.

## 1 Introduction

The mental health of healthcare professionals is critical to the resilience of the public health system (1). As a core component of the healthcare workforce, nurses experiencing high anxiety levels not only jeopardize their own well-being but also pose a significant threat to patient safety and care quality, making this a pressing public health issue (2). Globally, stress related to clinical practice affects approximately one-third of nurses (3). Tasked with providing high-quality care, nurses face considerable psychological stressors, including long working hours, shift rotations, exposure to patient suffering, and workplace violence (4, 5). These demands render nurses more susceptible to depression, anxiety, and burnout compared to other professional groups (6), ultimately impairing work efficiency, clinical performance, and the stability of the healthcare system (7).

Anxiety is among the most prevalent psychological issues reported by clinical nurses (8). Recent studies highlight the global scale of this challenge: in the United States, approximately 30% of nurses experience moderate to severe anxiety (9), with burnout studies indicating anxiety as a major component affecting up to 41.5% of hospital nurses (10). Survey from Japan reveals anxiety symptoms in about 24.2% of nurses (11). In China, a recent study of 7,114 nurses found an anxiety prevalence of 29.03% (12), which escalated to 46% among 4,188 nurses surveyed amid the COVID-19 pandemic (13). This persistent anxiety crisis not only threatens nursing workforce stability but also challenges the sustainable development of healthcare systems worldwide, underscoring the urgent need to identify protective factors and intervention pathways.

Drawing on the stress-buffering model (14), we hypothesize that the effect of social support on anxiety is mediated by psychological resilience. The underlying rationale is that social support does not neutralize anxiety directly (15). Rather, we propose that it first fosters the development of psychological resilience. This internalized resource then empowers nurses to better manage stressors, thereby reducing anxiety. Social support refers to the psychological, informational, and material resources that individuals obtain through social connections, which may help them cope with stress (16). Evidence suggests that enhancing social support is an effective strategy for mitigating psychological symptoms, buffering occupational stress, and reducing emotional distress among healthcare workers (17, 18). For instance, one study indicated that nurses with lower levels of social support were nearly twice as likely to experience anxiety symptoms compared to those with adequate support (19). However, the specific mechanisms underlying this protective effect require further elucidation. Psychological resilience—defined as the capacity to adapt and recover from adversity (20)—is a well-established protective factor against anxiety and depression (21). Empirical studies confirm that resilience-focused interventions can significantly reduce anxiety and stress among nurses (22). Critically, social support and psychological resilience are closely associated. Beyond directly alleviating distress, social support may also enhance an individual’s ability to cope with stress by fostering psychological resilience (23). Research suggests a positive correlation between the two, with potential synergistic effects strengthening emotional protection (24). This suggests that psychological resilience may act as a potential mediator, indicating that the protective effect of social support against anxiety is partly achieved by enhancing psychological resilience.

Nevertheless, the interrelationships between these constructs merit deeper investigation. Social support is best understood as an external resource, in contrast to psychological resilience, which is an intrinsic characteristic (25). The theoretical pathway explored in this study therefore investigates how external social resources (i.e., social support) contribute to the accumulation of internal psychological resources (i.e., psychological resilience), thereby helping to preserve core mental health resources and reduce manifestations of anxiety.

This study proposes that psychological resilience mediates the relationship between social support and anxiety among nurses. We aim to verify this mediating effect, determine its type and magnitude, and provide evidence for targeted mental health interventions. The findings are expected to help nurses better manage workplace stress, enhance their psychological well-being, and improve their overall quality of life.

## 2 Methods

### 2.1 Study design and participants

This cross-sectional survey was carried out from February to April 2025. The study population comprised registered clinical nurses with a minimum of 1 year of experience, directly engaged in patient care at hospitals in Chengdu, Sichuan Province, China. Individuals in purely administrative, teaching, or research roles were excluded from participation.

Participants were recruited through a process of stratified random sampling followed by snowball sampling: hospitals in Chengdu’s five main urban districts were first stratified by region and then by type (comprehensive, specialized, community) to select six representative hospitals, from which eligible nurses were subsequently recruited via snowball sampling.

The minimum required sample size was calculated *a priori* using the formula for cross-sectional studies: $n=\frac{Z^{2}\;P(1-P)}{d^{2}}$ (26). Based on a preliminary survey (*n* = 100) indicating an anxiety prevalence (*P*) of 35%, and with Z (the Z-statistic for a 95% confidence interval) set at 1.96, the margin of error (*d*) was set to 15% of *P* (*d* = 0.0525). This calculation yielded a minimum required sample size of 833 participants.

An online survey was administered through hospital-affiliated QQ and WeChat groups. Participants received a detailed description of the study’s purpose, along with assurances of anonymity and confidentiality, before providing informed consent. Of the 1,166 questionnaires collected, 33 were excluded due to incomplete data on the primary scales (anxiety, social support, psychological resilience) or key demographic variables, resulting in 1,133 valid responses for the final analysis.

### 2.2 Measurement scales

#### 2.2.1 Measurement of anxiety

The anxiety levels of nursing staff were assessed using the Self-Rating Anxiety Scale (SAS), a widely recognized instrument developed by Zung (27). It includes 20 items: nervousness, fear, panic, a sense of losing control, a premonition of misfortune, hand and foot trembling, headache, fatigue, restlessness, palpitations, dizziness, a feeling of fainting, dyspnea, paresthesia of the hands and feet, stomachache and indigestion, frequent urination, hyperhidrosis, facial flushing, sleep disturbance, and nightmares. Each item is rated on a 4-point Likert scale ranging from 1 (“Rarely”) to 4 (“Most of the time”). Five items (5, 9, 13, 17, and 19) are reverse-scored. Before calculating the total score, responses to these items are recoded (1 → 4, 2 → 3, 3 → 2, 4 → 1). The sum of all 20 items yields the total anxiety score, with a score above 40 indicating an anxious state. Higher scores reflect more severe anxiety symptoms. We report the Cronbach’s *α* for all scales in the Supplementary Figures S4–S6.

#### 2.2.2 Measurement of social support

Social support was assessed using the Social Support Rating Scale (SSRS) (28), which is based on the 27-item Social Support Questionnaire (SSQ) developed by Sarason et al. (29) and has been adapted to be more suitable for the Chinese context. The scale consists of 10 items, which are categorized into three dimensions: objective support (3 items), subjective support (4 items), and utilization of social support (3 items). The total score ranges from 12 to 66 points: scores ≤22 indicate low social support, scores between 23 and 44 indicate moderate social support, and scores >44 indicate high social support. The higher the score, the higher the level of social support.

#### 2.2.3 Measurement of psychological resilience

Nurses’ psychological resilience was assessed using the Connor-Davidson Resilience Scale (CD-RISC). Although the original scale contains 25 items and has demonstrated high reliability (Cronbach’s *α* = 0.89) (30), exploratory factor analysis conducted during our preliminary survey supported a four-factor structure more consistent with the characteristics of the Chinese sample (Supplementary Figures S1–S3). Accordingly, the scale was interpreted using four dimensions: self-efficacy, resiliency, optimism, and hope. Items were rated on a 5-point Likert scale ranging from 0 (“never”) to 4 (“almost always”). Total scores range from 0 to 100, with higher scores indicating greater psychological resilience.

### 2.3 Statistical methods

After preliminary assessment via histograms confirmed that anxiety, social support, and psychological resilience scores were approximately normally distributed, these variables were described using means and standard deviations (Mean ± SD). Differences in anxiety levels across categorical variables—such as age, years of nursing experience, professional title, education level, and hospital level—were examined using one-way analysis of variance (ANOVA), while an independent samples *t*-test was used to compare anxiety between nurses who worked night shifts and those who did not. Following significant ANOVA results, post-hoc pairwise comparisons were examined using Bayesian 95% credible intervals. Separately, Pearson’s correlation analysis was used to evaluate the bivariate correlations among anxiety, social support, and psychological resilience. Structural Equation Modeling (SEM) was conducted using AMOS 28.0.

To examine the mediating role of psychological resilience in the relationship between social support and anxiety, a series of linear regression models were constructed following the three-step procedure outlined by Baron and Kenny (31). The significance and type of mediation were further verified using the bootstrap method (32). All analyses were conducted in IBM SPSS Statistics 25.0 with the PROCESS macro (v4.0) by Hayes (33, 34), adopting a two-tailed significance level of *α* = 0.05.

## 3 Results

### 3.1 Demographic characteristics

The study included 1,133 nursing staff with a mean age of 25.8 ± 4.4 years (range: 18–60). All participants had at least 1 year of nursing experience, with a mean duration of 4.2 ± 3.6 years (range: 1–20). The sample’s anxiety level, measured by the SAS, showed a mean score of 38.67 ± 8.16. Among the participants, 404 (35.7%) met the criteria for an anxious state (SAS score ≥ 40). The distribution of anxiety scores across demographic characteristics is presented in Table 1.

**Table 1:** Distribution of anxiety scores among Chinese nurses (*n* = 1,133).

Individual characteristics		Sample size, *n*	Participants with anxiety, *n* (%)	Anxiety score, x̅ ± s	Lower bound^†^	Upper bound	*F*/*t*	*p*
Gender	Male	230	124 (53.9)	41.04 ± 8.24	39.99	42.08	4.963	<0.001*
	Female	903	280 (31)	38.07 ± 8.04	37.55	38.60		
Age (years)	<22	71	36 (50.7)	42.62 ± 10.06	40.73	44.51	6.042	<0.001*
	[22, 25)	421	149 (35.4)	38.29 ± 9.19	37.52	39.07		
	[25, 30)	458	168 (36.7)	38.53 ± 7.54	37.79	39.28		
	≥30	183	51 (27.9)	38.37 ± 5.61	37.20	39.55		
Nursing experience (years)	<3	459	179 (39)	40.05 ± 8.89	39.31	40.79	8.041	<0.001*
	[3, 5)	301	94 (31.2)	37.28 ± 8.67	36.36	38.19		
	[5, 10)	232	91 (39.2)	37.99 ± 6.43	36.95	39.03		
	≥10	141	40 (28.4)	38.32 ± 6.27	36.98	39.66		
Professional title	Primary	883	286 (32.4)	38.07 ± 8.20	37.54	38.60	11.419	<0.001*
	Intermediate	225	108 (48)	40.92 ± 7.72	39.87	41.98		
	Senior	25	10 (40)	39.80 ± 7.14	36.63	42.97		
Education	Secondary nursing education or below	31	5 (16.1)	37.65 ± 5.12	34.77	40.52	0.292	0.747
	Associate degree	668	235 (35.2)	38.76 ± 8.07	38.14	39.38		
	Bachelor’s degree or above	434	164 (37.8)	38.62 ± 8.48	37.85	39.39		
Hospital level	Grade 3 (3A/3B)	728	215 (29.5)	37.41 ± 7.83	36.83	37.98	30.027	<0.001*
	Grade 2 (2A/2B)	190	111 (58.4)	42.17 ± 8.88	41.04	43.30		
	Other	215	78 (36.3)	39.88 ± 7.53	38.82	40.95		
Night shift duty	Yes	896	332 (37.1)	38.73 ± 8.43	38.21	39.28	0.553	0.58
	No	237	72 (30.4)	38.41 ± 7.05	37.37	39.45		
Marital status	Unmarried/divorced	732	274 (37.4)	38.76 ± 8.54	38.16	39.35	0.211	0.646
	Married	401	130 (32.4)	38.52 ± 7.43	37.72	39.32		
Parental status	One child	203	66 (32.5)	37.67 ± 7.25	36.54	38.79	2.439	0.063
	Two children	73	25 (34.2)	38.96 ± 5.44	37.09	40.83		
	Married, no children	125	40 (32)	40.15 ± 8.79	38.72	41.58		
	Unmarried, no children	732	273 (37.3)	38.67 ± 8.48	38.08	39.26		

^†^Posterior 95% credible interval from Bayesian estimates of coefficients.

As shown in Table 1, gender, age, nursing experience, professional title, and hospital level were significantly associated with anxiety levels among nurses (all *p* < 0.05). Male nurses exhibited nearly 3 points higher anxiety scores than female nurses, with a 23-percentage-point greater prevalence of anxiety. Post-hoc pairwise comparisons detected a statistically significant difference only for nurses aged <22 years, who reported higher anxiety than all other age groups. No other inter-group differences were significant.

A similar pattern was observed for nursing experience: nurses with less than 3 years of experience reported significantly higher anxiety than their more experienced counterparts. In terms of professional title, nurses with intermediate titles showed the highest anxiety levels, significantly exceeding those with primary titles. Additionally, nurses in Grade 2 hospitals reported anxiety scores nearly 5 points higher than those in Grade 3 hospitals and approximately 3 points higher than those in other hospitals, such as community hospitals.

Although not statistically significant in this sample, descriptive trends suggested that married nurses without children had one of the highest mean anxiety scores, whereas those with only one child had the lowest. The overall difference across reproductive status groups approached but did not reach statistical significance (*p* = 0.063). Further investigation with larger samples is warranted to validate these preliminary observations.

### 3.2 Correlations between anxiety and social support among Chinese nurses

The relationship between social support and anxiety among Chinese nurses was examined using correlation analysis (Table 2). Social support and its three dimensions were significantly negatively correlated with anxiety scores, indicating that the more social support nurses receive, the lower their anxiety levels will be. These results support the hypothesis that social support serves as a protective factor against anxiety. Among the dimensions, support utilization showed the strongest negative correlation with anxiety, followed by objective support and subjective support (all *p* < 0.05). The three dimensions of social support were moderately and positively correlated with each other (0.307 ≤ *r* ≤ 0.526, all *p* < 0.05), reflecting their interrelatedness within a comprehensive social support system.

**Table 2:** Correlations between nurses’ anxiety and social support.

Variables	Anxiety	Objective support	Subjective support	Support utilization	Total social support score
Anxiety	1	−0.277**	−0.126**	−0.306**	−0.283**
Objective support	−0.277**	1	0.427**	0.526**	0.831**
Subjective support	−0.126**	0.427**	1	0.307**	0.799**
Support utilization	−0.306**	0.526**	0.307**	1	0.703**
Total social support score	−0.283**	0.831**	0.799**	0.703**	1

**p < 0.001, overall correlation coefficient is statistically significant.

Furthermore, the total social support score was highly correlated with each dimension (*r* > 0.7), demonstrating good structural validity of the Social Support Rating Scale in this sample.

### 3.3 Correlations between anxiety and psychological resilience among Chinese nurses

Psychological resilience and its four dimensions—self-efficacy, hope, resiliency, and optimism—were all significantly negatively correlated with anxiety (all *p* < 0.001), supporting the hypothesis that resilience acts as a protective factor. Among the dimensions, resiliency showed the strongest correlation with anxiety (*r* = −0.285), followed by optimism (*r* = −0.239) and hope (*r* = −0.224), while self-efficacy had the weakest correlation (*r* = −0.186). Analysis of the scale’s internal structure revealed very high correlations among self-efficacy, hope, and optimism (0.933 ≤ *r* ≤ 0.955). In contrast, the resiliency dimension was only moderately correlated with the other three (0.278 ≤ *r* ≤ 0.302), suggesting it represents a distinct facet of psychological resilience in this sample.

The significant correlations demonstrated in Tables 2, 3 satisfy the preliminary conditions for testing a mediation effect, as both the independent variable (social support) and the mediator (psychological resilience) are significantly associated with the dependent variable (anxiety). We therefore proceeded to formally test the proposed mediation model.

**Table 3:** Correlations between nurses’ anxiety and psychological resilience.

Variables	Anxiety	Self-efficacy	Hope	Resiliency	Optimism	Total psychological resilience score
Anxiety	1	−0.186**	−0.224**	−0.285**	−0.239**	−0.260**
Self-efficacy	−0.186**	1	0.933**	0.302**	0.940**	0.963**
Hope	−0.224**	0.933**	1	0.278**	0.955**	0.963**
Resiliency	−0.285**	0.302**	0.278**	1	0.281**	0.464**
Optimism	−0.239**	0.940**	0.955**	0.281**	1	0.966**
Psychological resilience	−0.260**	0.963**	0.963**	0.464**	0.966**	1

**p < 0.001, overall correlation coefficient is statistically significant.

### 3.4 Correlation analysis between social support and psychological resilience among Chinese nurses

Table 4 presents the correlation results. A significant positive correlation was found between the total scores of social support and psychological resilience (*r* = 0.361, *p* < 0.001). Specifically, the correlation of total social support with the individual resilience dimensions, ranked in descending order of strength, was optimism, hope, self-efficacy, and resiliency (all *p* < 0.001). Furthermore, among the social support dimensions, their correlations with total psychological resilience showed an ascending order of strength: support utilization, objective support, and subjective support (all *p* < 0.001).

**Table 4:** Results of the correlation analysis between social support and psychological resilience.

Variables	Total social support score	Objective support	Subjective support	Support utilization	Total psychological resilience score	Self-efficacy	Hope	Resiliency	Optimism
Total social support score	1	0.831**	0.799**	0.703**	0.361**	0.327**	0.329**	0.231**	0.350**
Objective support	0.831**	1	0.427**	0.526**	0.318**	0.285**	0.282**	0.235**	0.299**
Subjective support	0.799**	0.427**	1	0.307**	0.228**	0.221**	0.203**	0.124**	0.225**
Support utilization	0.703**	0.526**	0.307**	1	0.328**	0.277**	0.318**	0.200**	0.324**
Total psychological resilience score	0.361**	0.318**	0.228**	0.328**	1	0.963**	0.963**	0.464**	0.966**
Self-efficacy	0.327**	0.285**	0.221**	0.277**	0.963**	1	0.933**	0.302**	0.940**
Hope	0.329**	0.282**	0.203**	0.318**	0.963**	0.933**	1	0.278**	0.955**
Resiliency	0.231**	0.235**	0.124**	0.200**	0.464**	0.302**	0.278**	1	0.281**
Optimism	0.350**	0.299**	0.225**	0.324**	0.966**	0.940**	0.955**	0.281**	1

**p < 0.001, overall correlation coefficient is statistically significant.

### 3.5 Analysis of the mediating role of psychological resilience

As presented in Table 5, three regression models were constructed to examine the relationships among the variables. Model 1 showed that, after controlling for other covariates, social support remained a significant negative predictor of anxiety scores among nurses (*β* = −0.313, *p* < 0.01). In Model 2, social support was found to be independently and positively associated with psychological resilience (*β* = 1.295, *p* < 0.01). Model 3 further revealed that psychological resilience had a significant negative effect on anxiety (*β* = −0.051, *p* < 0.01), while the direct effect of social support on anxiety, though still significant, decreased in magnitude from–0.313 to–0.247. These results suggest that psychological resilience partially mediates the relationship between social support and anxiety. The increase in R^2^ from 0.124 to 0.150 indicates that the inclusion of psychological resilience improved the explanatory power of the model. All key pathways—social support to psychological resilience and psychological resilience to anxiety—were statistically significant (*p* < 0.001).

**Table 5:** Results of multivariate analysis of nurses’ anxiety with mediating effects.

Variables	Anxiety (model 1)	Psychological resilience (model 2)	Anxiety (model 3)
Constant	55.102** (19.126)	114.574** (11.957)	60.973** (20.222)
Age (year)	−0.171 (−2.079)	−0.439 (−1.602)	−0.194 (−2.383)
Professional title	0.287** (1.734)	0.728* (1.321)	0.324** (1.986)
Hospital level	1.437** (4.757)	−7.931** (−7.894)	1.031** (3.369)
Nurses’ social support	−0.313** (−9.429)	1.295** (11.722)	−0.247** (−7.116)
Nurses’ psychological resilience			−0.051** (−5.798)
*R* ^2^	0.124	0.196	0.150
*F*	*F* = 22.801, *p* < 0.001	*F* = 39.098, *p* < 0.001	*F* = 24.732, *p* < 0.001

Values in the table represent regression coefficients (*β*), with *t*-values in parentheses.

Significance levels: *p < 0.05, **p < 0.01.

Path coefficients along with statistical inference results are summarized in Table 6. The total effect of social support on anxiety (without the mediator) yielded a 95% confidence interval (CI) of (−0.378, −0.248), which excludes zero. Similarly, the direct effect of social support on anxiety, after adjusting for psychological resilience, had a 95% CI of (−0.315, −0.179), also excluding zero. The indirect effect of social support on anxiety through psychological resilience was significant, with a 95% CI of (−0.080, −0.038) that does not include zero. These findings support a significant partial mediating role of psychological resilience in the relationship between social support and nurses’ anxiety. The mediation effect accounted for approximately 21.1% of the total effect (indirect effect/total effect = −0.066/–0.313 ≈ 21.1%).

**Table 6:** Test results for mediating effect sizes in this study.

Analysis path	Meaning	Effect size	95% confidence interval		z/*t*	*p*
			Lower limit	Upper limit		
Social support → psychological resilience → anxiety	Indirect effect	−0.066	−0.080	−0.038	−6.297	<0.001
Social support → psychological resilience	X → M	1.295	1.078	1.511	11.722	<0.001
Psychological resilience → anxiety	M → Y	−0.051	−0.069	−0.034	−5.798	<0.001
Social support → anxiety	Direct effect	−0.247	−0.315	−0.179	−7.116	<0.001
Social support → anxiety	Total effect	−0.313	−0.378	−0.248	−9.429	<0.001

The 95% confidence interval refers to the 95% bootstrap confidence interval (95% BootCI), with the bootstrap method specified as the percentile bootstrap method. X = social support (independent variable); M = psychological resilience (mediator); Y = anxiety (dependent variable).

**Figure 1:**
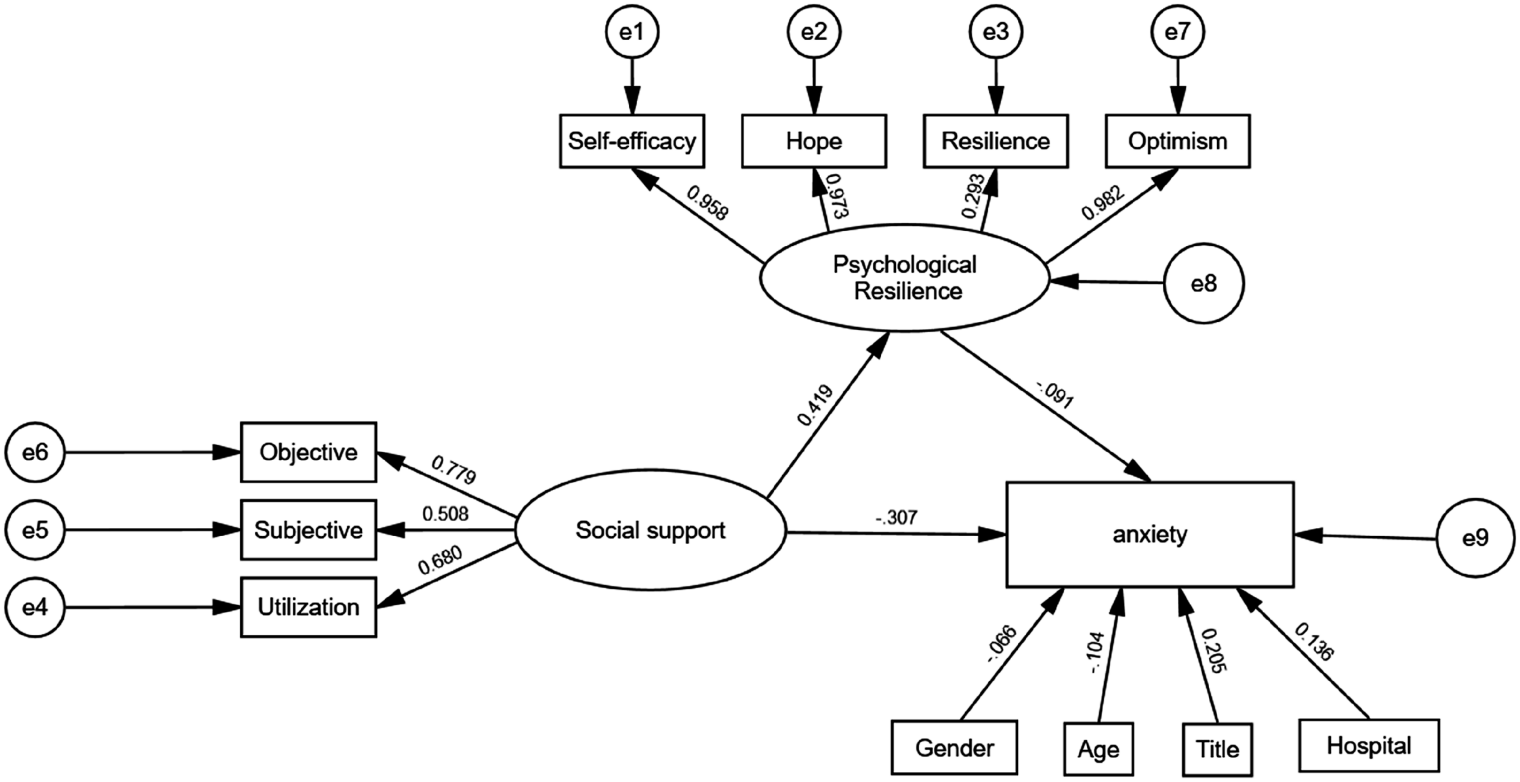
Path diagram of the relationships among social support, psychological resilience, and nurses’ anxiety.

The hypothesized mediation model was tested using structural equation modeling (SEM) with latent variables (Figure 1). To enable comparison across paths, standardized regression coefficients are presented. As all variables were modeled as latent factors, this approach accounts for measurement error. All path coefficients were significant (*p* < 0.05; Supplementary Table S1). The results indicate that, in addition to a direct negative effect on anxiety, social support had a significant positive effect on psychological resilience. Its effect on anxiety was also transmitted indirectly via this enhanced resilience. This latent-variable analysis confirms the findings from the observed-variable model, demonstrating result robustness.

**Figure 2:**
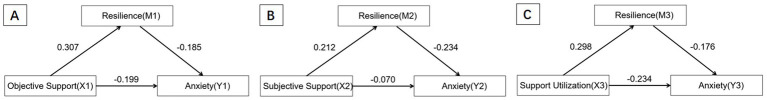
Psychological resilience mediates the relationship between three dimensions of social support and anxiety. **(A)** Schematic diagram of psychological resilience mediating the relationship between objective support and anxiety. **(B)** Schematic diagram of psychological resilience mediating the relationship between subjective support and anxiety. **(C)** Schematic diagram of psychological resilience mediating the relationship between utilization of support and anxiety.

To delineate the specific roles of each dimension, we decomposed the effects of the three social support facets within the mediation model (Figure 2; all paths significant per Supplementary Table S2). Analysis of the standardized path coefficients revealed a divergence: support utilization and objective support had relatively larger direct effects on anxiety, while the direct effect of subjective support was minimal. Notably, the pattern of indirect effects told a different story. The pathway mediated by psychological resilience was strongest for subjective support, with an indirect effect constituting 41% of its total impact.

## 4 Discussion

Grounded in a public health perspective, this study examines the interaction between social-environmental factors and individual characteristics in relation to nurses’ occupational mental health. Based on a cross-sectional survey in Chengdu, China, the findings support the hypothesized mediation model, wherein psychological resilience mediates the effect of social support on anxiety. This reveals a salient psychological mechanism among nursing staff: the pathway from the acquisition of external resources to the building of internal capacity, ultimately shaping emotional outcomes.

The primary finding confirms that psychological resilience mediates the effect of social support on anxiety. Conceptually, social support—an external resource encompassing connections with leaders, colleagues, family, and friends (35)—serves a dual function: it directly reduces anxiety, and it is also converted into psychological resilience. This internal resource, characterized by self-efficacy, hope, resiliency, and optimism (36), enhances nurses’ ability to deploy cognitive and emotional strategies under stress, thereby buffering against anxiety. This mechanism is not unique to our sample; it has been replicated in diverse populations. For example, Eliza et al. reported a significant mediation effect of psychological resilience (explaining up to 30.9% of the total effect) between social support and anxiety among nurses, foreign domestic helpers, and residents in Hong Kong (37). Consistent evidence from varied contexts supports this mechanism from nurses. First, in a study of people living with HIV/AIDS, psychological resilience fully mediated the social support-anxiety link, accounting for 68.42% of the effect (38). Second, research among postpartum women found that resilience partially mediated the relationship between increased support and reduced anxiety (39). Third, the COVID-19 pandemic underscored this pathway under crisis conditions; multiple studies confirmed that resilience mediated the effects of specific stressors—such as perceived pandemic stress, job stress, occupational exposure, and fear of infection—on the anxiety, depression, and distress of healthcare workers (40–43). Thus, psychological resilience emerges as a key mediator, defining a resource-transformation pathway: external support builds internal resilience, which ultimately safeguards emotional well-being. This pathway aligns with and elaborates upon two key theories. It concretely illustrates the COR Theory’s principle of resource investment and gain (44), showing how social support, as an external resource, translates into psychological resilience to prevent mental health depletion. It also provides a specific cognitive-personal mechanism for the Stress-Buffering Model (14), demonstrating that support alleviates anxiety by building resilience, thereby enacting the stress-buffering process.

Building on the confirmed partial mediation model, this study provides a nuanced understanding of how different dimensions of social support relate to anxiety. The pathways diverged markedly: while “support utilization” and “objective support” had stronger direct links to anxiety, “subjective support” exerted its influence predominantly indirectly through psychological resilience, with its mediated effect being nearly double. This underscores that the protective mechanism is not uniform; the subjective perception of available support is uniquely channeled through the development of internal resilience. This pattern diverges from findings in other contexts, such as among lung cancer patients, where resilience mediated the link for subjective support and support utilization but not for objective support (45), suggesting important population or context-specific moderators. Furthermore, a study of older adults in China demonstrated a gender difference: the association between subjective support and cognitive function was fully mediated by psychological resilience in men, whereas it was only partially mediated in women (46). Therefore, interventions should be population-specific. For the nursing population, our study yields two practical recommendations. First, reinforce the provision and accessible use of objective, material support. This involves securing necessary resources and training to utilize them effectively. Simultaneously, fostering a supportive climate that enhances nurses’ subjective perception and experience of support is critical, as this directly cultivates psychological resilience. Building this internal resource is key to safeguarding mental health and alleviating anxiety.

Our analysis revealed differential correlations between the constituent dimensions of psychological resilience—self-efficacy, hope, resiliency, and optimism (36)—and anxiety. Notably, resiliency (defined as focused persistence in adversity) (47) showed the strongest inverse association, highlighting the paramount importance of stable adaptability under stress for anxiety reduction. Similarly, optimism (48) and hope (49) were significantly negatively correlated with anxiety. Optimism fosters adaptive stress appraisal (50), whereas hope underpins the pursuit of viable pathways despite obstacles (49, 51), both mitigating anxiety. Of the four dimensions, self-efficacy exhibited the weakest, albeit significant, negative correlation. This likely reflects that while professional confidence facilitates proactive stress coping, its direct protective effect against anxiety may be less pronounced than that of other resilience facets.

It is also noteworthy that the anxiety levels observed among nurses in Chengdu, China, were relatively high, nearing the upper bound of prevalence estimates for Chinese nurses in a global synthesis (52). This elevated prevalence points to acute mental health strains within the current healthcare environment. The immediate context includes the legacy of the pandemic, specifically 3 years of lockdown measures in China. These pressures are compounded by longer-term structural factors, such as the ongoing economic slowdown and stricter healthcare payment reforms, which may adversely affect nurses’ socioeconomic stability and mental health (53). The high anxiety levels likely result from a confluence of acute, sub-acute, and chronic stressors. First, anxiety may be a lingering psychological sequel of the extreme stress nurses faced during the pandemic. Second, this is compounded by sustained post-pandemic pressures, as healthcare systems address care backlogs, potentially maintaining high workloads (54). Finally, these are exacerbated by the profession’s inherent, chronic stressors—including extended hours, high-intensity tasks, and shift-work disruption—which continuously elevate burnout risk and undermine mental health (55).

Policy and practice should focus on evidence-based interventions targeting psychological resilience—the critical mediator identified. For instance, individual-focused approaches like resilience training, mindfulness, and cognitive-behavioral therapy have proven effective (56). Simultaneously, healthcare institutions must implement system-level strategies, including mental health education, competency training, and confidential counseling. Implementing structured peer-support networks within clinical teams is also essential, as they facilitate mutual assistance and open communication, fostering team cohesion and sustained well-being (57, 58). This multi-pronged approach builds the core facets of resilience (self-efficacy, hope, resiliency, optimism), leading to significant anxiety reduction.

## 5 Limitations of the study

This study has several limitations. First, the cross-sectional design precludes causal inference regarding the relationships among social support, psychological resilience, and anxiety, so there may be confounding bias related to time. Second, as the sample was drawn exclusively from Sichuan Province, China, the generalizability of the findings to other regions or cultural contexts may be limited, there may be selection bias. Future research should adopt longitudinal or experimental designs to examine the long-term and causal effects of social support and resilience on anxiety. It would also be valuable to validate the current model in diverse healthcare systems and cultural settings. Furthermore, future studies should design, implement, and evaluate multi-level interventions based on the present findings, and conduct cost-effectiveness evaluations to inform public health resource allocation. Nurse characteristics were accounted for as confounders in this study and thus were not analyzed in detail. A fruitful direction for future research would be to conduct stratified analyses based on these or other clinically meaningful variables to uncover potential subgroup differences and elucidate nuanced intervention targets.

## 6 Conclusion

This empirical survey study, conducted among nurses in Chengdu, Sichuan Province, China, in the post-pandemic era, reveals two key findings. First, anxiety levels within the nursing cohort remain notably elevated. Second, psychological resilience plays a partial mediating role in the relationship between social support and anxiety. Notably, the strongest mediating effect of psychological resilience is observed on the pathway linking subjective support to anxiety. This suggests that the anxiety-alleviating function of social support may operate through the internalization of psychological resilience. Moreover, the interaction between perceived support from the social environment and individual nurse characteristics—such as psychological resilience—appears to be a critical factor in emotional regulation. The findings further indicate that, to advance public-health policy implementation through nurses’ occupational well-being, it is essential not only to foster a supportive environment but also to adopt systematic strategies that enhance nurses’ subjective perception of support, thereby cultivating their psychological resilience to mitigate anxiety.

## Data Availability

The raw data supporting the conclusions of this article will be made available by the authors, without undue reservation.

## References

[1] Søvold LE Naslund JA Kousoulis AA Saxena S Qoronfleh MW Grobler C . Prioritizing the mental health and well-being of healthcare workers: an urgent global public health priority. Front Public Health. (2021) 9:679397. doi: 10.3389/fpubh.2021.679397, 34026720 PMC8137852

[2] Li LZ Yang P Singer SJ Pfeffer J Mathur MB Shanafelt T. Nurse burnout and patient safety, satisfaction, and quality of care: a systematic review and meta-analysis. JAMA Netw Open. (2024) 7:e2443059. doi: 10.1001/jamanetworkopen.2024.43059, 39499515 PMC11539016

[3] Asefzadeh S Kalhor R Tir M. Patient safety culture and job stress among nurses in Mazandaran, Iran. Electron Physician. (2017) 9:6010. doi: 10.19082/6010, 29560154 PMC5843428

[4] Bautista JR Lauria PAS Contreras MCS Maranion MMG Abeleda RD. Specific stressors relate to nurses’ job satisfaction, perceived quality of care, and turnover intention. Int J Nurs Pract. (2020) 26:e12774. doi: 10.1111/ijn.12774, 31423700

[5] Starc J. Stress factors among nurses at the primary and secondary level of public sector health care: the case of Slovenia. Open Access Maced J Med Sci. (2018) 6:416–22. doi: 10.3889/oamjms.2018.100, 29531616 PMC5839460

[6] Lu G Xiao S He J Xie W Ge W Meng F . Prevalence of depression and its correlation with anxiety, headache and sleep disorders among medical staff in the Hainan Province of China. Front Public Health. (2023) 11:1122626. doi: 10.3389/fpubh.2023.1122626, 37441641 PMC10333496

[7] Pang Y Dan H Jung H Bae N Kim O. Depressive symptoms, professional quality of life and turnover intention in Korean nurses. Int Nurs Rev. (2020) 67:387–94. doi: 10.1111/inr.12600 32633425

[8] Chen J Liu X Wang D Jin Y He M Ma Y . Risk factors for depression and anxiety in healthcare workers deployed during the COVID-19 outbreak in China. Soc Psychiatry Psychiatr Epidemiol. (2021) 56:47–55. doi: 10.1007/s00127-020-01954-1, 32914298 PMC7483060

[9] Shanafelt T Ripp J Trockel M. Understanding and addressing sources of anxiety among health care professionals during the COVID-19 pandemic. JAMA. (2020) 323:2133–4. doi: 10.1001/jama.2020.5893 32259193

[10] Cimiotti JP Aiken LH Sloane DM Wu ES. Nurse staffing, burnout, and health care–associated infection. Am J Infect Control. (2012) 40:486–90. doi: 10.1016/j.ajic.2012.02.029, 22854376 PMC3509207

[11] Tahara M Mashizume Y Takahashi K. Coping mechanisms: exploring strategies utilized by Japanese healthcare workers to reduce stress and improve mental health during the COVID-19 pandemic. Int J Environ Res Public Health. (2020) 18:131. doi: 10.3390/ijerph18010131, 33375444 PMC7795636

[12] Chen F Zhao X Qian X Wang W Zhou Y Xu J. Relationships among sleep quality, anxiety, and depression among Chinese nurses: a network analysis. J Affect Disord. (2025) 389:119587. doi: 10.1016/j.jad.2025.119587, 40499830

[13] Peng P Liang M Wang Q Lu L Wu Q Chen Q. Night shifts, insomnia, anxiety, and depression among Chinese nurses during the COVID-19 pandemic remission period: a network approach. Front Public Health. (2022) 10:1040298. doi: 10.3389/fpubh.2022.1040298, 36544790 PMC9760836

[14] Cohen S Wills TA. Stress, social support, and the buffering hypothesis. Psychol Bull. (1985) 98:310. doi: 10.1037/0033-2909.98.2.310 3901065

[15] Yang L Wang N Li D Zhao X Wen M Zhang Y . Social support and anxiety, a moderated mediating model. Sci Rep. (2025) 15:29390. doi: 10.1038/s41598-025-14336-x 40790318 PMC12339740

[16] Cohen S. Social relationship and health. Am Psychol. (2004) 59:676–84. doi: 10.1037/0003-066X.59.8.676 15554821

[17] Labrague LJ De Los Santos JAA. COVID-19 anxiety among front-line nurses: predictive role of organisational support, personal resilience and social support. J Nurs Manag. (2020) 28:1653–61. doi: 10.1111/jonm.13121, 32770780 PMC7436313

[18] Cho SH. The influence of social support on the relationship between emotional demands and health of hospital nurses: a cross-sectional study. Healthcare. (2021) 9:115. doi: 10.3390/healthcare9020115, 33498995 PMC7912004

[19] Misganaw A Hailu M Bayleyegn G Aderaw M Yigzaw ZA Alemu T . Exploring factors affecting nurse anxiety in Northwest Ethiopia: a multicenter study. Front Psych. (2024) 15:1434701. doi: 10.3389/fpsyt.2024.1434701, 39415889 PMC11479901

[20] Norris FH Tracy M Galea S. Looking for resilience: understanding the longitudinal trajectories of responses to stress. Soc Sci Med. (2009) 68:2190–8. doi: 10.1016/j.socscimed.2009.03.043, 19403217

[21] Jeamjitvibool T Duangchan C Mousa A Mahikul W. The association between resilience and psychological distress during the COVID-19 pandemic: a systematic review and meta-analysis. Int J Environ Res Public Health. (2022) 19:14854. doi: 10.3390/ijerph192214854, 36429573 PMC9690093

[22] Abbasalizadeh M Farsi Z Sajadi SA Atashi A Fournier A. The effect of resilience training with mHealth application based on micro-learning method on the stress and anxiety of nurses working in intensive care units: a randomized controlled trial. BMC Med Educ. (2024) 24:442. doi: 10.1186/s12909-024-05427-w, 38658914 PMC11041025

[23] Parlak M Michelson D Easterbrook MJ. The mediating role of social support and resilience in the relationship between social identity and mental health among international students. BJPsych Open. (2025) 11:e118. doi: 10.1192/bjo.2025.72 40468827 PMC12188238

[24] Hou Y Zhang Y Cao X Lei G Liu G. The association between perceived social support and resilience among Chinese university students: a moderated mediation model. Psychol Sch. (2024) 61:1474–90. doi: 10.1002/pits.23122

[25] Wang Y Qiu Y Ren L Jiang H Chen M Dong C. Social support, family resilience and psychological resilience among maintenance hemodialysis patients: a longitudinal study. BMC Psychiatry. (2024) 24:76. doi: 10.1186/s12888-024-05526-4, 38279114 PMC10811847

[26] Pourhoseingholi MA Vahedi M Rahimzadeh M. Sample size calculation in medical studies. Gastroenterol Hepatol Bed Bench. (2013) 6:14–7. doi: 10.22037/ghfbb.v6i1.332 24834239 PMC4017493

[27] Zung WW. A rating instrument for anxiety disorders. Psychosomatics. (1971) 12:371–9. doi: 10.1016/S0033-3182(71)71479-0, 5172928

[28] Wang X Zhang F Ge Y Ding Y Liu T. The associations between social support, self-regulatory fatigue, and health-promoting behaviors among people with type 2 diabetes mellitus: a cross-sectional survey. Front Public Health. (2023) 11:1281065. doi: 10.3389/fpubh.2023.1281065, 38155890 PMC10752976

[29] Sarason IG Levine HM Basham RB Sarason BR. Assessing social support: the social support questionnaire. J Pers Soc Psychol. (1983) 44:127. doi: 10.1037/0022-3514.44.1.127

[30] Connor KM Davidson JR. Development of a new resilience scale: the Connor-Davidson resilience scale (CD-RISC). Depress Anxiety. (2003) 18:76–82. doi: 10.1002/da.10113, 12964174

[31] Baron RM Kenny DA. The moderator-mediator variable distinction in social psychological research: conceptual, strategic, and statistical considerations. J Pers Soc Psychol. (1986) 51:1173–82. doi: 10.1037//0022-3514.51.6.1173, 3806354

[32] Davison AC Hinkley DV. Bootstrap methods and their application. Cambridge: Cambridge University Press (1997).

[33] Hayes AF. Partial, conditional, and moderated moderated mediation: quantification, inference, and interpretation. Commun Monogr. (2018) 85:4–40. doi: 10.1080/03637751.2017.1352100

[34] Hayes AF. Introduction to mediation, moderation, and conditional process analysis: a regression-based approach. New York, NY: Guilford Publications (2017).

[35] House JS. Work stress and social support. Boston: Addison-Wesley (1981).

[36] Lorenz T Beer C Pütz J Heinitz K. Measuring psychological capital: construction and validation of the compound PsyCap scale (CPC-12). PLoS One. (2016) 11:e0152892. doi: 10.1371/journal.pone.0152892, 27035437 PMC4817957

[37] Wong ELY Qiu H Sun KS Mo PKH Lai AHY Yam CHK . Social support, resilience, and mental health among three high-risk groups in Hong Kong: a mediation analysis. Int J Public Health. (2024) 69:1606828. doi: 10.3389/ijph.2024.1606828, 38681117 PMC11045880

[38] Sun YB Song B Zhen C Zhang C Cheng J Jiang TJ. The mediating effect of psychological resilience between social support and anxiety/depression in people living with HIV/AIDS-a study from China. BMC Public Health. (2023) 23:2461. doi: 10.1186/s12889-023-17403-y, 38066520 PMC10709980

[39] Ben-David H Miller KJ. Resilience mediates the relationship between social support and anxiety in postpartum mothers. Clin Psychol. (2025) 29:119–30. doi: 10.1080/13284207.2025.2462842

[40] Yildirim M Arslan G. Perceived risk and mental health problems among healthcare professionals during COVID-19 pandemic: exploring the mediating effects of resilience and coronavirus fear. Int J Ment Heal Addict. (2020) 20:1035–45. doi: 10.1007/s11469-020-00424-8 PMC7668285 33223977

[41] Shi LS Xu RH Xia Y Chen DX Wang D. The impact of COVID-19-related work stress on the mental health of primary healthcare workers: the mediating effects of social support and resilience. Front Psychol. (2021) 12:800183. doi: 10.3389/fpsyg.2021.800183, 35126252 PMC8814425

[42] Yang R Ke Q Chan SW Liu Y Lin H Li W . A cross-sectional examination of the relationship between nurses’ experiences of skin lesions and anxiety and depression during the COVID-19 pandemic: exploring the mediating role of fear and resilience. J Nurs Manag. (2022) 30:1903–12. doi: 10.1111/jonm.13638, 35434883 PMC9115287

[43] Maggi G Baldassarre I Barbaro A Cavallo ND Cropano M Nappo R . Mental health status of Italian elderly subjects during and after quarantine for the COVID-19 pandemic: a cross-sectional and longitudinal study. Psychogeriatrics. (2021) 21:540–51. doi: 10.1111/psyg.12703, 33955115 PMC8242477

[44] Hobfoll SE. The influence of culture, community, and the nested-self in the stress process: advancing conservation of resources theory. Appl Psychol. (2001) 50:337–70. doi: 10.1111/1464-0597.00062

[45] Hu T Xiao J Peng J Kuang X He B. Relationship between resilience, social support as well as anxiety/depression of lung cancer patients: a cross-sectional observation study. J Cancer Res Ther. (2018) 14:72–7. doi: 10.4103/jcrt.JCRT_849_17, 29516963

[46] Zhang Y Wu Y Li Y. Sex differences in the mediating effect of resilience on social support and cognitive function in older adults. Geriatr Nurs. (2023) 53:50–6. doi: 10.1016/j.gerinurse.2023.06.013, 37429110

[47] Southwick SM Bonanno GA Masten AS Panter-Brick C Yehuda R. Resilience definitions, theory, and challenges: interdisciplinary perspectives. Eur J Psychotraumatol. (2014) 5:25338. doi: 10.3402/ejpt.v5.25338 PMC4185134 25317257

[48] Scheier MF Carver CS. Dispositional optimism and physical well-being: the influence of generalized outcome expectancies on health. J Pers. (1987) 55:169–210. doi: 10.1111/j.1467-6494.1987.tb00434.x, 3497256

[49] Edwards ME Booker JA Cook K Miao M Gan Y King LA. Hope as a meaningful emotion: hope, positive affect, and meaning in life. Emotion. (2025) 25:1365–80. doi: 10.1037/emo0001513, 40111802

[50] Kim EY Chang SO. Exploring nurse perceptions and experiences of resilience: a meta-synthesis study. BMC Nurs. (2022) 21:26. doi: 10.1186/s12912-021-00803-z, 35042488 PMC8766352

[51] Onieva-Zafra MD Fernández-Muñoz JJ Fernández-Martínez E García-Sánchez FJ Abreu-Sánchez A Parra-Fernández ML. Anxiety, perceived stress and coping strategies in nursing students: a cross-sectional, correlational, descriptive study. BMC Med Educ. (2020) 20:1–9. doi: 10.1186/s12909-020-02294-z PMC7574568 33081751

[52] Mahmud S Hossain S Muyeed A Islam MM Mohsin M. The global prevalence of depression, anxiety, stress, and, insomnia and its changes among health professionals during COVID-19 pandemic: a rapid systematic review and meta-analysis. SSRN Electron J. (2021) 7:e07393. doi: 10.1016/j.heliyon.2021.e07393 PMC8261554 34278018

[53] Oyat FWD Oloya JN Atim P Ikoona EN Aloyo J Kitara DL. The psychological impact, risk factors and coping strategies to COVID-19 pandemic on healthcare workers in the Sub-Saharan Africa: a narrative review of existing literature. BMC Psychol. (2022) 10:284. doi: 10.1186/s40359-022-00998-z, 36457038 PMC9714392

[54] Ledesma JR Chrysanthopoulou SA Lurie MN Drph JBN Papanicolas I. Health system resilience during the COVID-19 pandemic: a comparative analysis of disruptions in care from 32 countries. Health Serv Res. (2024) 59:e14382. doi: 10.1111/1475-6773.14382 39295092 PMC11622287

[55] Mehta A Awuah WA Ng JC Kundu M Yarlagadda R Sen M . Elective surgeries during and after the COVID-19 pandemic: case burden and physician shortage concerns. Ann Med Surg. (2022) 81:104395. doi: 10.1016/j.amsu.2022.104395 PMC9388274 35999832

[56] Liu J Wei S Qiu G Li N Wang D Wu X . Relationship between rumination and post-traumatic growth in mobile cabin hospital nurses: the mediating role of psychological resilience. Prev Med Rep. (2023) 34:102266. doi: 10.1016/j.pmedr.2023.102266, 37288138 PMC10241969

[57] Roberts NJ McAloney-Kocaman K Lippiett K Ray E Welch L Kelly C. Levels of resilience, anxiety and depression in nurses working in respiratory clinical areas during the COVID pandemic. Respir Med. (2021) 176:106219. doi: 10.1016/j.rmed.2020.106219, 33248362 PMC7648185

[58] Orgambídez-Ramos A de Almeida H. Work engagement, social support, and job satisfaction in Portuguese nursing staff: a winning combination. Appl Nurs Res. (2017) 36:37–41. doi: 10.1016/j.apnr.2017.05.012, 28720237

