# Supplementary material for: Exploring the mediating role of psychological resilience between social support and anxiety in nurses: a cross-sectional study in Chengdu, Sichuan, China
Source: Front Public Health. 2026 Feb 23;14:1765061. doi: 10.3389/fpubh.2026.1765061 (PMC12968000; doi:10.3389/fpubh.2026.1765061)

**Supplementary data：**

Exploring the Mediating Role of Psychological Resilience Between Social Support and Anxiety in Nurses: A Cross-Sectional Study in Chengdu, Sichuan, China

Junyi Hou^1†^, Mingzhu Song^1†^, Hui Li^2^, Xinlin Miao^1^, Xia Wang^1^,

Yesha Liu^3^, Jianfeng Sun^2*^ and Chan Huang^1*^

^1^School of Preclinical Medicine & School of Nursing, Chengdu University, Chengdu, 610106, P. R. China

^2^School of Acupuncture and Moxibustion, Affiliated Hospital of Chengdu University of TCM, Chengdu 611731, P. R. China

^3^Hospital of Chengdu University of Traditional Chinese Medicine (TCM Hospital of Sichuan Province), Chengdu, 610040, P. R. China

† Junyi Hou and Mingzhu Song have contributed equally to this work and share first authorship.

*** Correspondence:**Huang Chan and Sun Jianfeng are the co-corresponding authors.
huangchan @cdu.edu.cn (Huang Chan)

**Table S1 Regression Weights in Structural Equation Modeling**

| **Path relationship** | **Estimate** | **S.E.** | **t** | **P** | **Standized Estimate** |
| --- | --- | --- | --- | --- | --- |
| Psychological Resilience <= Social Support | 2.311 | 0.204 | 11.346 | <0.001 | **0.419** |
| Self-efficacy <= Psychological Resilience | 1 | / | / | / | 0.958 |
| Hope <= Psychological Resilience | 1.039 | 0.012 | 86.484 | <0.001 | 0.973 |
| Resilience <= Psychological Resilience | 0.19 | 0.019 | 10.193 | <0.001 | 0.293 |
| Optimism <= Psychological Resilience | 1.036 | 0.011 | 91.668 | <0.001 | 0.982 |
| Utilization <= Social Support | 1 | / | / | / | 0.680 |
| Subjective <= Social Support | 1.236 | 0.091 | 13.618 | <0.001 | 0.508 |
| Objective <= Social Support | 1.664 | 0.104 | 15.954 | <0.001 | 0.779 |
| Anxiety <= Social Support | -1.639 | 0.202 | -8.097 | <0.001 | **-0.307** |
| Anxiety <= Psychological Resilience | -0.088 | 0.03 | -2.876 | 0.004 | **-0.091** |
| Anxiety <= Title | -0.192 | 0.05 | -3.865 | <0.001 | -0.104 |
| Anxiety <= Age | 3.515 | 0.462 | 7.612 | <0.001 | 0.205 |
| Anxiety <= Hospital | 1.405 | 0.278 | 5.047 | <0.001 | 0.136 |
| Anxiety <= Gender | -1.339 | 0.548 | -2.444 | 0.015 | -0.066 |

**Table S2** **Psychological resilience mediates the relationship between three dimensions of social support and anxiety**

| **Effect decomposition of the three social support dimensions** | | | | **Estimate** | **S.E.** | **t** | **p** | **Standized Estimate** | **Proportion of Mediated Effect** |
| --- | --- | --- | --- | --- | --- | --- | --- | --- | --- |
| **Objective Support** | **Total Effect** |  |  | -0.636 | 0.070 | -9.103 | <0.001 | -0.255 |  |
|  | Direct Effect | Objective Support => Anxiety | c1 | -0.495 | 0.073 | -6.828 | <0.001 | **-0.199** |  |
|  | **Indirect Effect** | Objective Support => Resilience | a1 | 2.660 | 0.238 | 11.162 | <0.001 | 0.307 |  |
|  |  | Resilience => Anxiety | b1 | -0.053 | 0.009 | -6.188 | <0.001 | -0.185 |  |
|  |  |  | a1*b1 | -0.141 | 0.025 | (-0.193,-0.097) ^†^ | | -0.057 | 22.2% |
| **Subjective Support** | **Total Effect** |  |  | -0.263 | 0.064 | -4.100 | <0.001 | -0.120 |  |
|  | Direct Effect | Subjective Support => Anxiety | c2 | -0.154 | 0.064 | -2.412 | 0.016 | **-0.070** |  |
|  | **Indirect Effect** | Subjective Support => Resilience | a2 | 1.611 | 0.218 | 7.380 | <0.001 | 0.212 |  |
|  |  | Resilience => Anxiety | b2 | -0.067 | 0.009 | -7.918 | <0.001 | -0.234 |  |
|  |  |  | a2*b2 | -0.108 | 0.019 | (-0.148,-0.073) ^†^ | | -0.050 | 41.0% |
| **Support Utilization** | **Total Effect** |  |  | -1.038 | 0.101 | -10.286 | <0.001 | -0.286 |  |
|  | Direct Effect | Support Utilization => Anxiety | c3 | -0.848 | 0.104 | -8.125 | <0.001 | **-0.234** |  |
|  | **Indirect Effect** | Support Utilization => Resilience | a3 | 3.744 | 0.348 | 10.758 | <0.001 | 0.298 |  |
|  |  | Resilience => Anxiety | b3 | -0.051 | 0.009 | -5.962 | <0.001 | -0.176 |  |
|  |  |  | a3*b3 | -0.191 | 0.035 | (-0.262,-0.128) ^†^ | | -0.052 | 18.4% |

Note: ^†^Bootstrapt 95%CI

**Figure S1-S3 Exploratory factor analysis of the CD-RISC.**


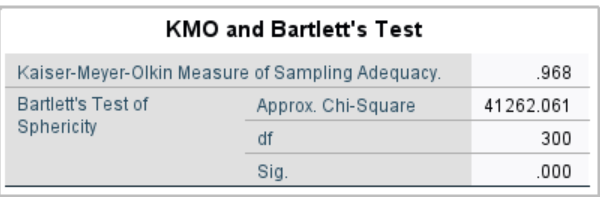


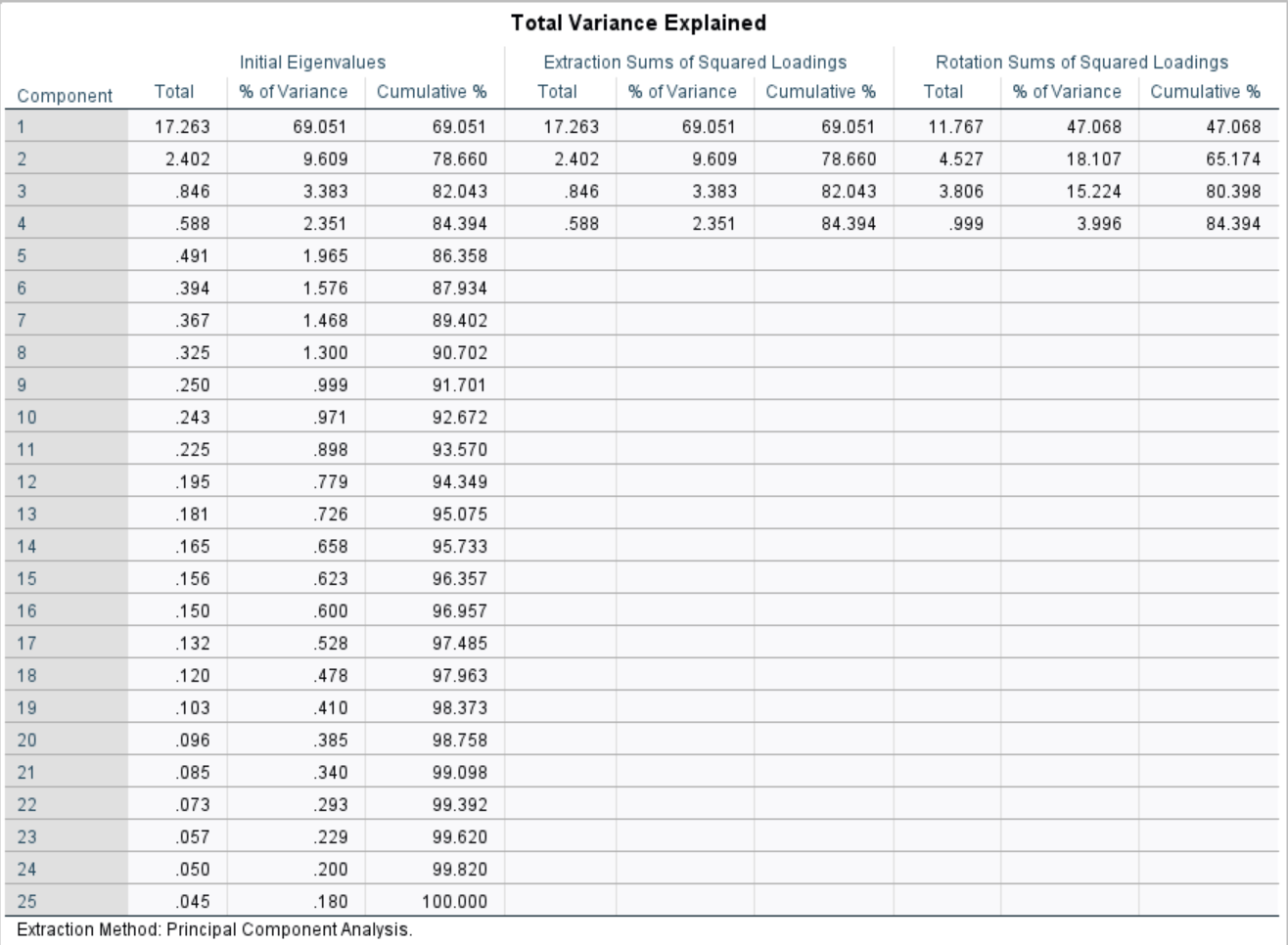

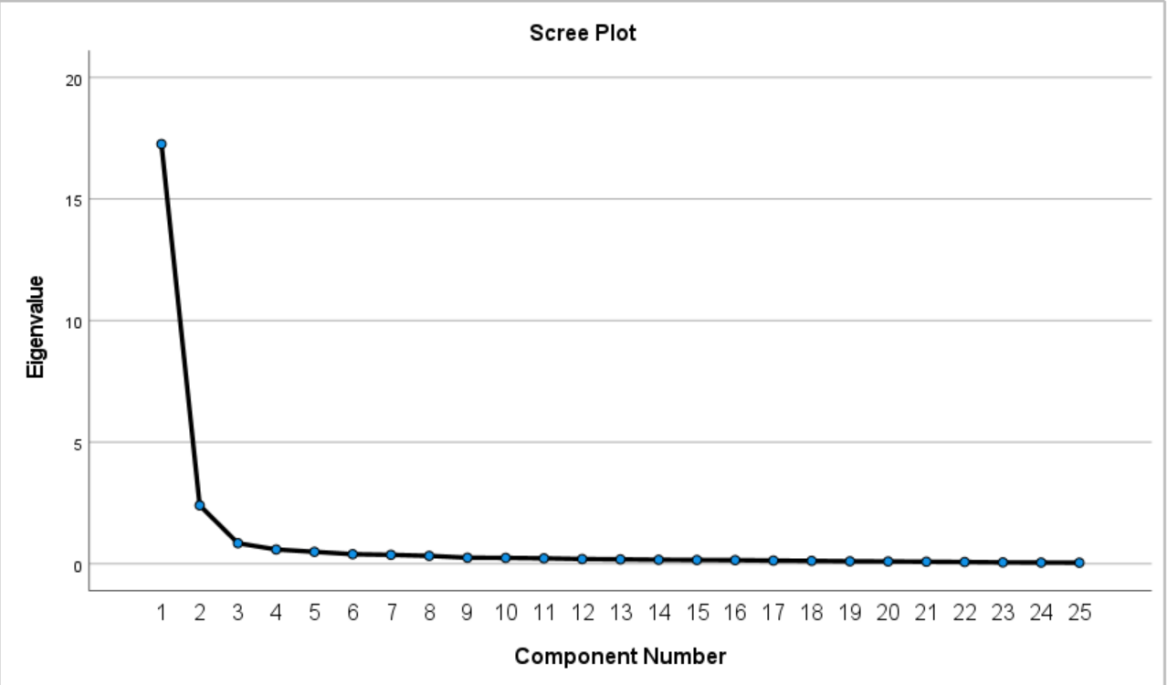


**Figure S4-S6** **Cronbach’s alpha coefficients for all scales**


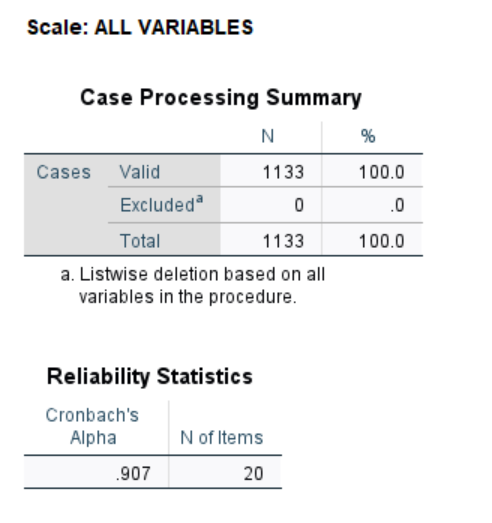

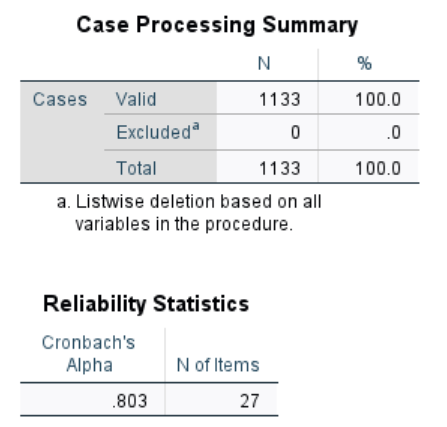

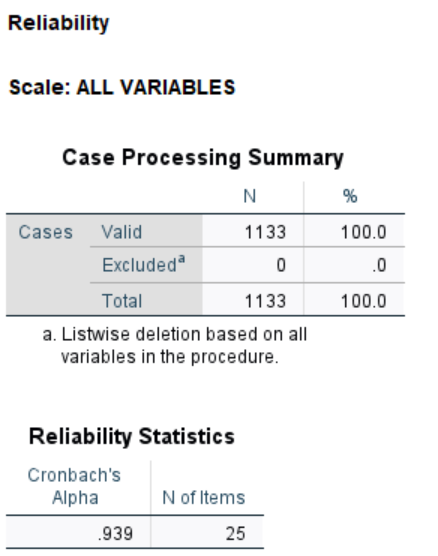

Supplement: Supplementary file 1 [file Table_1.docx]
